# Medical Student Knowledge of Neglected Tropical Diseases in Peru: A Cross-Sectional Study

**DOI:** 10.1371/journal.pntd.0004197

**Published:** 2015-11-02

**Authors:** Renato A. Errea, George Vasquez-Rios, Jorge D. Machicado, Maria Susana Gallardo, Marilhia Cornejo, Jorge F. Urquiaga, Diego Montoya, Rodrigo Zamudio, Angelica Terashima, Luis A. Marcos, Frine Samalvides

**Affiliations:** 1 Facultad de Medicina Alberto Hurtado, Universidad Peruana Cayetano Heredia, Lima, Peru; 2 Instituto de Medicina Tropical Alexander von Humboldt, Universidad Peruana Cayetano Heredia, Lima, Peru; 3 Division of Gastroenterology, Hepatology and Nutrition, University of Pittsburgh Medical Center, Pittsburgh, Pennsylvania, United States of America; 4 Division of Infectious Diseases, Department of Medicine, Stony Brook University, Stony Brook, New York, United States of America; Universidad Nacional Autónoma de México, MEXICO

## Abstract

In developing countries, education to health-care professionals is a cornerstone in the battle against neglected tropical diseases (NTD). Studies evaluating the level of knowledge of medical students in clinical and socio-demographic aspects of NTD are lacking. Therefore, a cross-sectional study was conducted among students from a 7 year-curriculum medical school in Peru to assess their knowledge of NTD by using a pilot survey comprised by two blocks of 10 short questions. Block I consisted of socio-demographic and epidemiological questions whereas block II included clinical vignettes. Each correct answer had the value of 1 point. Out of 597 responders (response rate: 68.4%), 583 were considered to have valid surveys (male:female ratio: 1:1.01; mean age 21 years, SD ± 2.42). Total knowledge showed a raising trend through the 7-year curriculum. Clinical knowledge seemed to improve towards the end of medical school whereas socio-demographic and epidemiological concepts only showed progress the first 4 years of medical school, remaining static for the rest of the curricular years (p = 0.66). Higher mean scores in socio-demographic and epidemiological knowledge compared to clinical knowledge were seen in the first two years (p<0.001) whereas the last three years showed higher scores in clinical knowledge (p<0.001). In conclusion, students from this private medical school gained substantial knowledge in NTD throughout the career which seems to be related to improvement in clinical knowledge rather than to socio-demographic and epidemiological concepts. This study assures the feasibility of measuring the level of knowledge of NTD in medical students and stresses the importance of evaluating education on NTD as it may need more emphasis in epidemiological concepts, especially at developing countries such as Peru where many people are affected by these preventable and treatable diseases.

## Author Summary

Since the promulgation of the United Nations Millennium Development Goals in 2000 and the posterior creation of the WHO’s Department of Control of Neglected Tropical Diseases five years later; encouraging progress in the diagnosis, treatment and prevention of these infectious diseases has been made. However, millions of people are still affected. Efforts to control NTD should come from different instances: from the high policy-decision makers to the young health-care and science professionals. Countries should consider to evaluate the quality of the medical training in order to develop and sustain efficient control programs of NTD, under the assumption that adequate education and knowledge from health-care providers are key starting points, especially in endemic areas. Having said that, universities—uniquely positioned to provide biomedical, epidemiological and clinical expertise- play an important role. Therefore, this study provides an initial approach in measuring NTD knowledge in medical students and should encourage the development of further surveys that may help to evaluate existing NTD education programs, especially because current global health problems could benefit from students’ awareness at early stages of medical training.

## Introduction

The concept of neglected tropical diseases (NTD) is a set of disabling conditions that are the most common chronic infections in the poorest people [1,2]. These conditions are typically prevalent in low-income communities in rural or pre-urban areas that lack of sanitation and access to health care [3]. The NTD represent an additional worrisome factor to these poor populations because they may cause several detrimental consequences alongside other socio-economic issues that already affect them. Some of the NTD may cause permanent impairment by restricting a person the ability to work or gain proper education [4].

The impact of NTD in public health around the world is notorious. About 1.2 billion people are infected by NTD [5] and over 350 million people are already disabled or severely impaired by them [6]. The situation is more striking in developing countries such as in Latin America and the Caribbean (LAC), where approximately 40% (of the estimated 556 million) of people live below the poverty line [7,8] and the total disease burden (measured in disability-adjusted life years -DALYs) of NTD may exceed those caused by malaria, tuberculosis or HIV/AIDS [5]. For these reasons, the control and elimination of NTD are now recognized as priorities within the Millennium Development Goals (MDGs) and it also targets for sustainable poverty reduction [9, 10].

In Peru, 23.9% and 4.7% of people live in poor and extreme poor conditions, respectively [11], and several NTD are considered endemic such as geohelminthiasis, fascioliasis, strongyloidiasis, cysticercosis, echinococcosis, Chagas disease, dengue and leishmaniasis [12]. Thus, the number of people infected or at risk of being infected by any of these NTD is worrisome. Hence, the development of control programs for these diseases in Peru are mandatory, and several research groups have dedicated efforts to study these diseases [12].

A great part of the effort to control NTD is to be recognized readily by the clinician in the field, who should manage an adequate level of knowledge of them. Therefore, the familiarity with these diseases should begin during medical school training. To the best of our knowledge, studies evaluating the level of knowledge of NTD of medical students are lacking. Thus, it is important to know if the current curricular strategies in medical schools at developing countries fulfill the clinical and epidemiological needs of these vulnerable populations. Therefore, the objective of this study is to provide a first approach to measure the knowledge level of NTD among medical students from a private medical school at Lima, Peru.

## Methods

### Study design

A cross-sectional survey was administered using a written questionnaire to medical students registered in one medical school in Lima, Peru.

### Instrument

The questionnaire was developed to gather information regarding knowledge in NTD from medical students currently enrolled in a medical school. Since no previous questionnaires or surveys were found in the literature after a thorough research in large databases (including LAC databases) such as Pubmed, Hinari, Proquest, EMBASE, Scielo, LILACS and Google Scholar, we designed a twenty multiple-choice questionnaire in the participant’s native language. The questionnaire focused on the most prevalent NTD in LAC [13], excluding other diseases only present in non-LAC countries. The questionnaire was divided in two parts. The first part (block I) consisted of ten short questions designed to ask respondents general data about socio-demographic and epidemiological impact concepts of NTD. The second part (block II), questions number 11 to 20, consisted of clinical vignettes with a maximum of two-sentence extension per question. Answers were developed so that they only included one best answer. Each correct answer had the value of 1 point, making a maximum score of 20.

### Study population

A total of 852 students are part of the School of Medicine Alberto Hurtado at Universidad Peruana Cayetano Heredia in Lima, Peru. Students enrolled in this private medical school were invited to participate in the study. This school of medicine contains a 7 year-program and has strong research programs in tropical medicine and infectious diseases [14]. The first three years are dedicated to study general and biomedical sciences. Both 4^th^ and 5^th^ years consist of clinical clerkships. In the last 2 years of training (6th and 7th years), the medical student gains experience in clinical practice as an active intern. We invited to participate all students 18 years old or older enrolled in the school of medicine. We excluded students in medical leave or absenteeism. Students who agreed to participate and gave oral informed consent, were included in the analysis.

### Intervention

Students were informed that the questionnaire was anonymous and that the results would not have any consequences in their curricular notes. After giving oral consent and voluntarily accepting to participate, the questionnaire sheet was delivered to the participants and a limit of ten minutes was given to complete the questionnaire. The intervention lasted seven days throughout the entire medical school. A questionnaire was considered invalid when one or both parts had more than 50% unanswered questions.

### Statistical analysis

A database was performed in Microsoft Excel. Results were analyzed using STATA version 12 (StataCorp.College Station, TX, USA) and were considered to be statistically significant when p<0.05. Mean scores year by year were calculated and comparisons were performed using t-test analysis with two-tailed p values and ANOVA.

### Ethical considerations

The protocol was presented and approved by the Institutional Review Board (IRB) of Universidad Peruana Cayetano Heredia, Lima, Peru. An oral informed consent was given and confidentiality was assured for all the information provided. Personal identifiers were not included on questionnaires.

## Results

Out of 852, a total of 597 medical students participated in the study (response rate 68.4%). The most common causes of refusal to participate in the study were the lack of time to complete the questionnaire and feeling tired at the time when the students were approached. In addition, 14 were considered as invalid questionnaires and were not included in the analysis, making a final number of 583 participants who had valid questionnaires. The mean age was 21 years (SD ± 2.42). By gender, the distribution was in a male:female ratio of 1:1.01.

**Fig 1:**
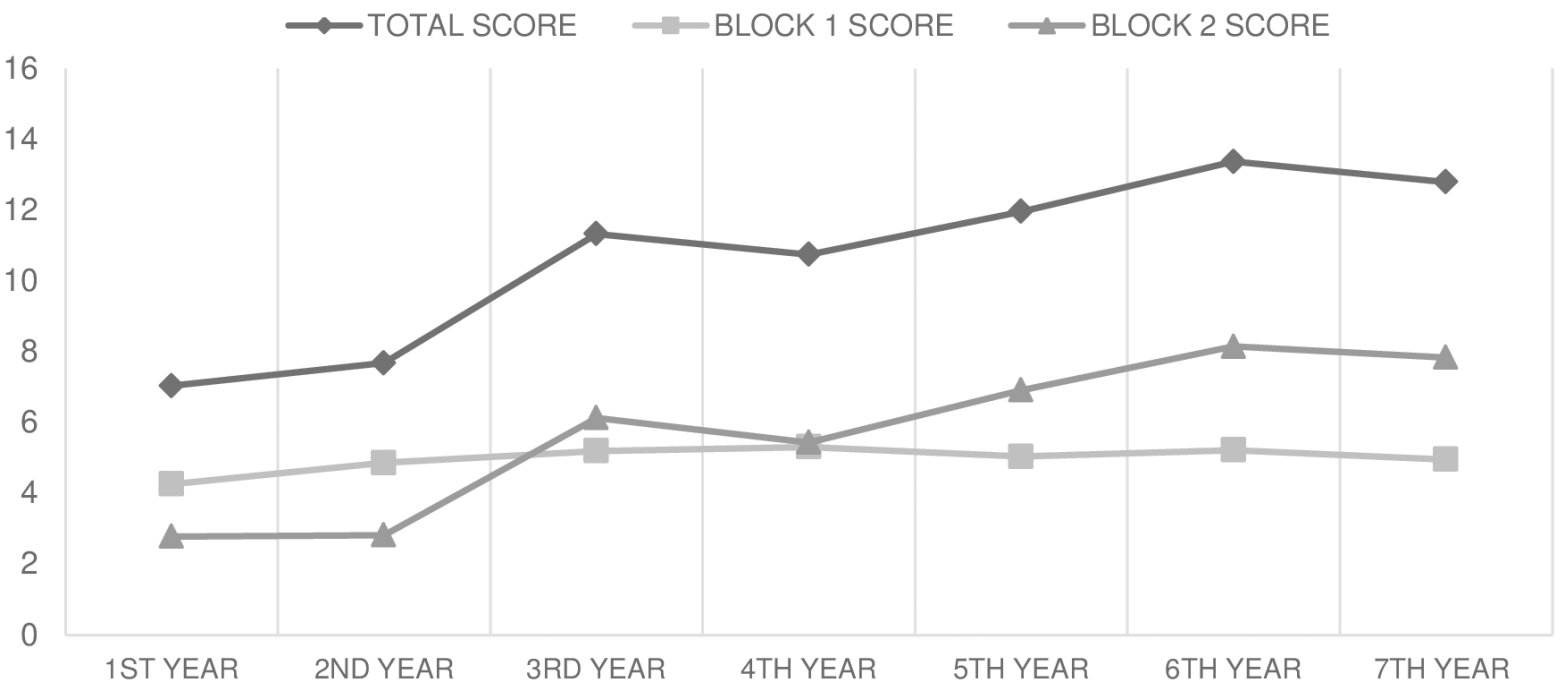
Trend of mean scores of total knowledge, socio-demographic and epidemiological knowledge (block I) and clinical knowledge (block II) by year of study at medical school. ANOVA statistics for (A) “Total score”, p<0.001 (B) “Block I score”, p<0.001 (C) “Block II score”, p<0.001 (D) Separately analysis of block I: “Block I score from 1^st^ to 4^th^ year”, p<0.001; “Block I score from 4^th^ to 7^th^ year”, p = 0.66.

Overall, there was a trend to an increase in the mean score of total knowledge through the years of study (Fig 1). The mean score of total knowledge was higher in the last year of the career compared to the mid-year (4^th^ year, p<0.05), and this one higher than the first year’s mean score (p<0.001).

However, when analyzed scores from both blocks separately, clinical knowledge showed a raising trend compared to socio-demographic and epidemiological knowledge which only showed some progress the first 4 years of medical school, remaining static for the rest of the curricular years (ANOVA, p = 0.66) (Fig 1). When a comparison of the mean scores was done year by year, the first two curricular years showed higher mean scores in socio-demographic and epidemiological knowledge than in clinical knowledge (p<0.001); the fourth year did not show significant difference (p = 0.68); while those in the fifth, sixth and seventh years showed higher scores in clinical knowledge (p<0.001).

## Discussion

In the present study, we found a progressively significant increase in the knowledge on NTD of medical students throughout the 7 curricular years. This is an encouraging finding and may suggest that the majority of students from this medical school graduate with an overall optimal knowledge of diseases included in the NTD set.

On the other hand, interesting results came up by analyzing separately block I and II. We found higher mean scores in socio-demographic and epidemiological impact knowledge compared to clinical knowledge among students from the first years (1^st^ and 2^nd^) of medical school (p<0.001) whereas the opposite occurred in students from later years (5^th^, 6^th^ and 7^th^; p<0.001). Furthermore, socio-demographic and epidemiological knowledge did not raise from the 4^th^ to 7^th^ year of the career (p = 0.66). This may indicate that clinical knowledge at this medical school improves towards the end of the career whereas the epidemiological concepts remain stable.

Improvement in clinical knowledge can be explained by the time of initiation of clinical courses in this medical school, which typically start at 4^th^ year including a specific Infectious Diseases clerkship during the 5^th^ year. Besides, students from clinical courses and clerkships have access to a major national referral tropical medical center (The Institute of Tropical Medicine *Alexander von Humboldt*), being exposed to a high number of patients with an infectious and/or tropical disease seeking care at this referral center. This data suggest clinical NTD training is probably being conducted adequately in this medical school.

On the other hand, the unchanged socio-demographic and epidemiological knowledge in the second half of the career may indicate that the concepts gained in the first years remained the same for the rest of the career. The early acquisition of socio-demographic and epidemiological knowledge may have occurred during the two complementary courses of public health and one of epidemiology dictated during the 1^st^ and 2^nd^ year. Moreover, one might expect that knowledge continues raising further throughout the career due to the Infectious Diseases clerkship at 5^th^ year, which includes education on epidemiology in addition to clinical concepts of infectious diseases. However, the results from this study did not show this trend, which suggest that the concepts in the knowledge of socio-demographic and epidemiological concepts may need revision.

As the fight against NTD needs an integrated understanding and a multidisciplinary approach, both the knowledge in clinical and socio-epidemiological factors should be interconnected from early stages of a healthcare provider career. In this study, the lack of progression in epidemiological knowledge may need to be addressed in different ways including participation and commitment from students and medical schools. For instance, some attempts to improve overall education on NTD include the introduction of courses or seminars targeted to NTD [5, 15, 16], local research studies carried out by students with university financial support [12, 17–20]; and participation in international training programs based on field research activities which become “twinning” opportunities between developed and developing countries [21, 22].

Despite the innovative character of this survey, limitations of the study are the lack of inclusion of other students from other medical schools and the lack of a previous standardized questionnaire to compare our results properly. Also, the type of questions might may not necessarily test for all epidemiologic, socio-demographic and clinical knowledge about NTD. Another limitation of this study is that approximately one third of students refused to participate to complete the questionnaire. However, the reason of refusal was due to fatigue or lack of time; thus, the score results of the group of students who completed the questionnaire at the end of the study were likely more accurate. Although other studies should confirm the validity of our findings, the present study shows that measuring knowledge of NTD in medical students is feasible.

In conclusion, students from this private medical school in Peru have gained substantial knowledge in NTD throughout their 7-year curriculum which seems to be related to improvement in clinical knowledge rather than to socio-demographic and epidemiological concepts. These results stress the importance of measuring the level of knowledge of NTD in undergraduate medical students in order to evaluate the need of intensifying the education in epidemiological concepts, providing to future medical doctors optimal tools to cope with these preventable and treatable diseases from a public and global health perspective.
